# The Plant Growth-Promoting Ability of Alfalfa Rhizobial Strains Under Nickel Stress

**DOI:** 10.3390/microorganisms13020340

**Published:** 2025-02-05

**Authors:** Mila Pešić, Sonja Tošić Jojević, Biljana Sikirić, Vesna Mrvić, Marina Jovković, Mira Milinković, Snežana Andjelković, Olivera Stajković-Srbinović

**Affiliations:** 1 Institute of Soil Science, Teodora Drajzera 7, 11000 Belgrade, Serbia; pesicmila@yahoo.com (M.P.); soils.tosic@gmail.com (S.T.J.); soils.sikiric@gmail.com (B.S.); soils.mrvic@gmail.com (V.M.); jovkovic.marina.90@gmail.com (M.J.); miramilinkovic@yahoo.com (M.M.); 2 Institute for Forage Crops Kruševac, Globoder, 37251 Kruševac, Serbia; snezana.andjelkovic@ikbks.com

**Keywords:** *Medicago sativa*, *Sinorhizobium*, trace metals, nickel, plant growth promotion

## Abstract

The growth and nutrient balance of legumes can be disrupted in soils with increased nickel (Ni) concentrations. The inoculation of legumes with rhizobia, symbiotic nitrogen-fixing bacteria, can be used for the alleviation of trace metal stress in plants. This study evaluated the Ni tolerance of alfalfa rhizobia isolates and some plant growth-promoting traits in the presence of Ni: indole-3-acetic acid (IAA) production, Ni biosorption potential, and the effect of rhizobia on alfalfa (*Medicago sativa* L.) growth. The strains were characterized as *Shinorhizobium meliloti*, *Sinorhizobium medicae*, and *Rhizobium tibeticum*. In total, 70% of the tested strains tolerate up to 0.8 mM Ni, while 15% of the strains tolerate 1.2 mM Ni. The production of IAA was maintained in the presence of Ni until bacterial growth was stopped by raising the Ni concentration. Alfalfa seed germination is significantly reduced in the presence of 0.5 mM Ni, while a significant reduction in 10-day-old seedling length already occurs at a Ni concentration of 0.03 mM. In the plant experiment, when alfalfa was inoculated with rhizobial strains, nodulation was maintained up to 0.05 mM Ni, but a significant reduction in nodule number was detected at 0.01 mM Ni. At the concentration of 0.005 mM Ni, inoculation with 12 particular rhizobial strains significantly improved the number of nodules per plant, plant height, and root length, as well as plant shoot dry weight, compared to non-inoculated plants with Ni addition. However, higher concentrations caused a reduction in all of these plant growth parameters compared to the plants without Ni. The selected rhizobia strains showed a Ni biosorption capacity of 20% in the in vitro assay. The inoculation of alfalfa with effective rhizobial strains improves growth parameters compared to non-inoculated plants in the presence of certain concentrations of Ni.

## 1. Introduction

In certain concentrations, trace metals are naturally present in the soils. However, their continuous accumulation in soil is becoming a growing problem, especially in agricultural soils. One of the most significant pollutants is nickel (Ni), which is released into the environment both by natural and human activities [1].

In soils with elevated Ni concentrations, the growth and nutrient composition of different plant species can be disrupted, and in high concentrations, Ni is toxic and hazardous to plants, animals, and humans. Nickel is an essential micronutrient for higher plants and some bacteria, a cofactor of the urease enzyme, and other enzymes in bacteria such as NiFe-hydrogenase, some superoxide dismutases, etc. [2]. The reaction catalyzed by urease, an enzyme widely distributed in various groups of organisms, is the hydrolysis of urea to ammonia and carbonic acid as final products [3]. In leguminous plants, NiFe-hydrogenase recycles hydrogen, a by-product of nitrogen reduction in nitrogen fixation by rhizobia in legumes [4]. Excessive Ni in plants negatively influences photosynthesis, respiration, and mineral nutrition, while toxic effects include reductions in dry matter production and yield [5]. Legumes can generally accumulate high amounts of Ni, which is important for improved urease activity, enhanced biological nitrogen fixation (BNF), N (protein) accumulation, and increasing organic acid content. Since trace metal pollution in soil is a significant threat to livestock as well as human beings via the food chain [6], it is essential to take appropriate measures to lower the risk of their accumulation in fodder crops.

Plant growth-promoting rhizobacteria (PGPR) can alleviate the toxicity of trace metals by promoting plant growth and by affecting the bioavailability of metals in soils [7]. PGPR enhance plant growth in the presence of trace metals in soil through improved plant nutrition, nitrogen fixation, phosphate solubilization, indole-3-acetic acid (IAA) production, ACC (1-aminocyclopropane-1-carboxylic acid) deaminase activity, iron acquisition, etc. PGPR lower the bioavailability of metals in soils through metal biosorption, bioaccumulation, redox reaction, mobilization, precipitation, and transformation [7]. In the process of phytostabilization, pollutants in the soil are immobilized either as a result of their absorption and accumulation in the plant roots and/or by bacteria [8]. Some of the most utilized bacteria in trace metal stress alleviation are *Pseudomonas*, *Bacillus*, *Azospirillum*, *Azotobacter*, *Serratia*, *Rhizobium*, etc., species able to promote the growth of various plants in metal-contaminated environments [9].

Legumes of the genus *Medicago* are important forage crops with established cultivation techniques which have also been investigated for their application in phytoremediation [10]. Alfalfa (*Medicago sativa* L.) is one of the most cultivated species in the world, having diverse ecological and physiological advantages; it successfully adapts to different soils and climate conditions. Alfalfa can establish effective nitrogen-fixing symbiosis with rhizobia from the genus *Sinorhizobium* (syn. *Ensifer*) with the amount of N_2_ usually fixed by alfalfa in the field varying between 140 and 210 kg ha^−1^ year^−1^ [11]. Besides nitrogen fixation, inoculation with the *S. meliloti* and *S. medicae* strains results in the promotion of some metal bioaccumulation within root nodules in contaminated soils [12,13]. Further, the inoculation of alfalfa with *Sinorhizobium* and other PGPR mainly increases the retention of the majority of the metals in the root zone, and consequently, the plant shoots are safe for use [14]. Besides nitrogen fixation, many rhizobia are producers of plant hormones, which promote plant growth, the secretion of acids and enzymes that solubilize phosphates, etc. In addition, rhizobia can act on metals directly in the soil by chelation, precipitation, transformation, biosorption, and accumulation and thus reduce metal toxicity. Nodules containing high concentrations of rhizobia can also serve as biosorbents or storage for metals [15]. In addition, the safety of rhizobia for humans and animals has been demonstrated after decades of legume inoculation, making them optimal for inoculant development. Investigations of soils from different parts of Serbia have shown the frequent presence of alfalfa in conditions of a high total content of trace metals, that is, above MAC, especially Cr, Pb, Ni, and As [16]. Alfalfa for animal feed is often grown near highways, mines, and other sources of pollution, so the potential for trace metals to enter the food chain is significant. Considering that, as is the case with other legumes, alfalfa is able to absorb a considerable amount of Ni, and Ni concentrations in plants increase significantly in both shoots and roots along with the Ni concentration in the soil [17], special attention is required in alfalfa cultivation in such soils. On the other hand, some findings imply that other leguminous plants such as soybean, horse gram, faba bean, etc., associated with specific rhizobial strains could be used to lower Ni in plants when grown in Ni-contaminated soils [18,19,20]. Hence, the use of Ni-tolerant alfalfa rhizobia inoculants would be of great importance for lowering the risk of Ni accumulation in alfalfa grown in such soils. Although there are some studies regarding various trace metals, to the best of our knowledge, limited information is available regarding alfalfa and Ni stress alleviation by rhizobia.

The objective of this study was to examine the possibility of using rhizobial strains to promote growth in alfalfa plants in conditions with elevated Ni concentrations. The hypothesis was that the rhizobial strains tolerant to Ni with symbiotic potential can improve the growth of alfalfa under Ni stress. Firstly, the alfalfa rhizobial strains were tested for Ni tolerance, and their plant growth-promoting traits under elevated Ni concentrations were evaluated (IAA production, biosorption capacity). The strains were genetically characterized and screened for metal resistance genes. For the most performant strains, the effect on alfalfa plant growth in a medium with rising Ni concentrations was evaluated. The goal was to obtain strains capable of promoting the growth and development of alfalfa under Ni stress, representing promising candidates for further testing in the sustainable management of Ni-contaminated soil.

## 2. Materials and Methods

### 2.1. Alfalfa Rhizobial Strains and Culture Conditions

The rhizobial strains specific for alfalfa (*Sinorhizobium* spp. and *Rhizobium* sp.) used in this study belong to the Collection of Bacteria of the Institute of Soil Science, Belgrade [21], or are new isolates from this study (Table S1). The strains were grown in yeast mannitol agar medium (YMA) for rhizobia [22].

### 2.2. Isolation of Alfalfa Rhizobia from Soil Samples

Isolation of alfalfa rhizobia from soil samples was performed using the method of plant infection by dilutions of soil suspension [23]. For plant cultivation, sterile Jensen’s nitrogen-free medium was used in the culture tubes. Seeds of *Medicago sativa* were surface sterilized by 96% alcohol, and after that with 0.2% HgCl_2_, and thoroughly washed 5–6 times using sterile distilled water. Dilutions of the soil suspension of 10^−1^, 10^−2^, 10^−3^, and 10^−4^ were made with a sterile physiological solution. Seven-day-old seedlings were inoculated with 0.5 mL of decimal dilutions of the tested soil. The plants were grown under controlled conditions in a light chamber, with a 16 h day/8 h night regime. The plants were grown for 6 weeks. The presence of rhizobia nodulating alfalfa in the examined soils was determined visually, by the presence of nodules on the roots of plants inoculated with soil suspensions. From the nodulated plants, root nodules were collected, surface sterilized by 96% ethanol for a few seconds and then in 0.1% HgCl_2_ solution (3–5 min), and rinsed with sterilized water 5–6 times [23]. The nodules were crushed in a physiological solution and streaked on YMA plates with Congo red. The plates were incubated for 2–3 days at 28 °C. Bacterial colonies were selected according to the morphology and restreaked to obtain a pure bacterial culture. Pure Gram-negative isolates were reinoculated in alfalfa to confirm the rhizobial status.

### 2.3. Nickel Tolerance of Alfalfa Rhizobia

The preliminary screening of isolates’ ability to grow in YMA medium supplemented with NiSO_4_ × 7H_2_O (0, 0.005, 0.01, 0.03, 0.05, 0.07, 0.08, 1.00, 1.10, 1.20 mM) was performed in Petri dishes by the replica plating method [22]. Isolates were grown in yeast mannitol broth medium (YMB) for 48 h, after which OD600 nm was adjusted to be 1, and 20 µL of each adjusted culture was applied to Petri dishes containing the abovementioned increasing concentrations of NiSO_4_ × 7H_2_O. Petri dishes without the addition of Ni were used as a control. Inoculated Petri dishes were incubated at 28 °C for 3 days, and the presence of bacterial growth was recorded.

In order to confirm the results obtained on Petri dishes, the tolerance to the same NiSO_4_ × 7H_2_O concentrations was determined in YMB for selected isolates, while the growth of bacteria was determined quantitatively. First, 1 mL of each overnight rhizobial culture (OD600 nm = 0.4) was inoculated in 20 mL of YMB containing concentrations of NiSO_4_ × 7H_2_O chosen based on the experiment with Petri dishes (0, 0.1, 0.4, or 0.7 mM) and incubated at 28 °C and 150 rpm on the orbital shaker, for 72 h, in triplicates. The OD of each inoculum was measured at 600 nm using a spectrophotometer after 72 h of incubation. The test was performed in triplicates, and mean values were presented.

### 2.4. The IAA Production by Rhizobial Strains in the Presence of Nickel

The ability of bacterial isolates to produce IAA was tested in YMB medium without L-tryptophan or supplemented with 2 mg ml^−1^ of L-tryptophan as well as with 0, 0.05, 0.1, 0.4, or 0.7 mM NiSO_4_ × 7H_2_O. Bacterial cultures grown in YMB for 72 h were centrifuged, and 1 mL of supernatant was mixed with 2 mL of Salkowski reagent (0.01 mM of FeCl_3_ in 35% HClO_4_). The concentration of IAA was measured after 25 min by a spectrophotometer at 530 nm [24]. A standard curve obtained from standard concentrations of pure IAA was used to determine the IAA concentration. The production of IAA was tested in six replicates for each isolate.

### 2.5. The ACC Deaminase Activity Test

The ACC deaminase activity was screened on a Dworkin and Foster (DF) medium [25] with the addition of ACC (final concentration 3 mM) as the only nitrogen source. Isolates were grown in mineral salt medium (glucose, 5 g; NaCl, 4 g; K_2_HPO_4_, 1.73 g; KH_2_PO_4_, 0.68 g; MgSO_4_ × 7H_2_O, 0.03 g; NH_4_NO_3_, 1 g; CaCl_2_ × 2H_2_O, 0.02 g per L of medium) for 72 h at 28 °C and 150 rpm. Then, 1.5 mL of the culture was centrifuged for 5 min at 10,000 rpm, the supernatant was discarded, and the pellet containing bacterial cells was washed using 1 mL of saline solution. The washing step was repeated 3 times. Subsequently, 20 µL of bacterial cells suspended in saline solution was spotted on the DF agar medium, and the plates were incubated for 7 days at 28 °C. Isolates that could grow on DF medium have the ability to use ACC as the only nitrogen source, and the positive result indicated the presence of ACC deaminase activity.

### 2.6. Nickel Biosorption Capacity of Rhizobial Strains

The capacity of rhizobia to adsorb nickel was tested in vitro. The strains were grown in Ni-supplemented YMB liquid media, and concentrations of Ni were determined using ICP-OES (Thermo iCAP 6300 Duo, Inductively Coupled Plasma Optical Emission Spectrometry, ICP-OES, Cambridge, UK). The nickel removal capacity of rhizobia was calculated based on a comparison of Ni concentrations in culture media and non-inoculated media.

Samples of fresh rhizobial cultures (10^8^ CFU mL^−1^) with a volume of 1 mL were inoculated in YMB medium supplemented with concentrations of 0, 0.1, or 0.4 mM Ni, and grown at 28 °C and 150 rpm for 3 days. The bacterial cultures were then centrifuged for 10 min at 5000 rpm, and the supernatant was collected and examined using ICP-OES. The YMB, with each of the tested concentrations of Ni and without inoculum, was used as a blank. The differences in Ni concentration between the inoculated and non-inoculated YMB suggested that the studied bacteria removed Ni from the medium, whether by accumulating it inside or on the surface of the cell. The experiment was repeated 3 times.

### 2.7. Alfalfa Seed Germination in the Presence of Nickel

A germination test was performed with Jensen medium [23] in Petri dishes. The medium was supplemented with increasing concentrations of Ni (0, 0.005, 0.01, 0.03, 0.05, 0.1, 0.5, 0.8, or 1 mM), and the percentage of germinated seeds and seedling length were determined after 10 days of incubation in the dark.

### 2.8. Rhizobial Strain Effect on Alfalfa Growth in the Presence of Nickel

The effect of selected rhizobial isolates on alfalfa growth (symbiotic efficiency) was evaluated in test tubes under controlled conditions. Seeds of alfalfa were surface sterilized, and one seed was put in a test tube with 30 mL of Jensen agar supplemented with increasing Ni concentrations (0.005 up to 0.4 mM Ni) [23]. The seeds were inoculated with 0.5 mL of bacterial suspension grown for 72 h, and the test tubes were kept in the light chamber. Two groups of control plants were used: plants without inoculation and without N supplementation—control plants Ø; plants supplemented with 0.05% KNO_3_ solution and without inoculation—control plants with nitrogen NØ. After eight weeks of sowing, the plants were sampled, when the inoculated plants should give a final differentiation of the effectiveness of strains. The number of root nodules, plant height, and root length were recorded for each treatment. After sampling, plant material was dried in an oven at 70 °C, and shoot (SDW) and root dry weight (RDW) were recorded. The inoculation was performed in seven replications, and mean values were presented.

### 2.9. Genetic Identification of the Strains

#### 2.9.1. Sequencing of 16S rDNA Region

Total DNA was isolated as described previously [20]. Genetic determination of strains up to the species level was performed using sequences obtained after Sanger sequencing of the 16S rRNA gene, which was amplified using two pairs of specific primers given in Table 1. Obtained overlapping sequences were aligned into a single contig. Sequencing services were performed by Macrogen Europe (Amsterdam, the Netherlands). 16S rDNA sequences of our strains, obtained after Sanger sequencing, were used as queries for retrieving DNA sequences with various percentages of homology from NCBI GenBank using nucleotide BLAST tool (https://blast.ncbi.nlm.nih.gov/ (accessed on 25 November 2024)). To ensure the reliability of the phylogenetic analysis, we downloaded sequences from reference strains only (American Type Culture Collection, Laboratorium voor Microbiologie Gent, National Reference Bacterial Collection, Center for Biological Research and Applied Sciences, China Center for Biological Agriculture and Microbiology). The multiple sequence alignment of the sequences downloaded from NCBI and sequences from this study was performed using MUSCLE, integrated in MEGA XI software [26,27]. The nucleotide substitution models were estimated by comparing the BIC (Bayesian Information Criterion) scores. The model with the lowest BIC value was considered most suitable for the maximum likelihood method (ML) and chosen for phylogenetic tree construction using MEGA XI software. A suitable parameter for rate among sites was selected based on the results of the model selection. For the phylogeny test, the bootstrap method with 1000 replications was selected. The ML tree was constructed using the Nearest Neighbor Interchange (NNI) heuristic algorithm.

#### 2.9.2. PCR Analysis of Selected Genetic Determinants

The detection of genetic determinants conferring tolerance to the metals Ni, Co, and Cd (*ncc*), Cr (*chr*), and Zn (*smt*) was performed by partial amplification of the loci in the *ncc,* chr, and *smt* operons and visualization of the PCR products on agarose gel. The presence of the ACC deaminase gene in the genome of the isolates was also determined. The primers used for amplification are given in Table 1 [28,29,30]. PCR programs were run under optimized conditions of amplification as summarized in Table 2. If the operons were present in the bacterial genome, fragments with lengths of 1,141, 450, and 507 base pairs for *nccA*, *chrB*, and *smtA*, respectively, were detected in 1% agarose gel electrophoresis and visualized by UV illumination after staining with 0.5 μg mL^−1^ ethidium bromide. If the ACC deaminase gene was present in the bacterial genome, a PCR product with a length of 800 base pairs was detected.

### 2.10. Statistical Analysis

The effect of the treatments on bacterial and plant parameters was evaluated using analysis of variance (ANOVA; statistical program, SPSS 22 program), and the Duncan multiple range test was used to test differences between means. All data represented are the mean ± standard deviation (SD) of particular replicates of each treatment. Correlations between parameters were also analyzed.

## 3. Results

### 3.1. Alfalfa Rhizobia Tolerance to Ni

**Figure 1:**
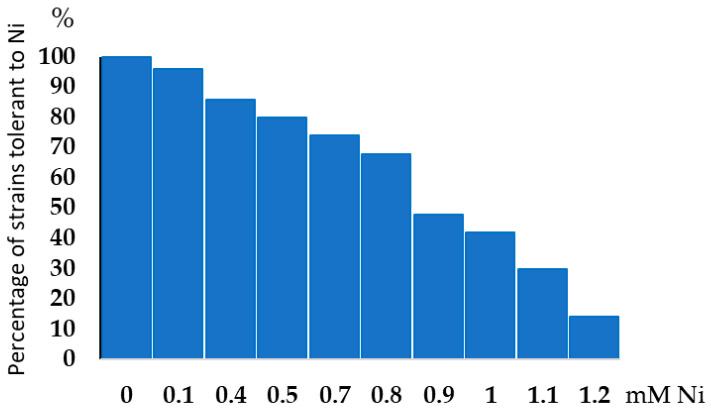
Percentage of strains tolerant to particular Ni concentrations with respect to total number of strains tested.

**Figure 2:**
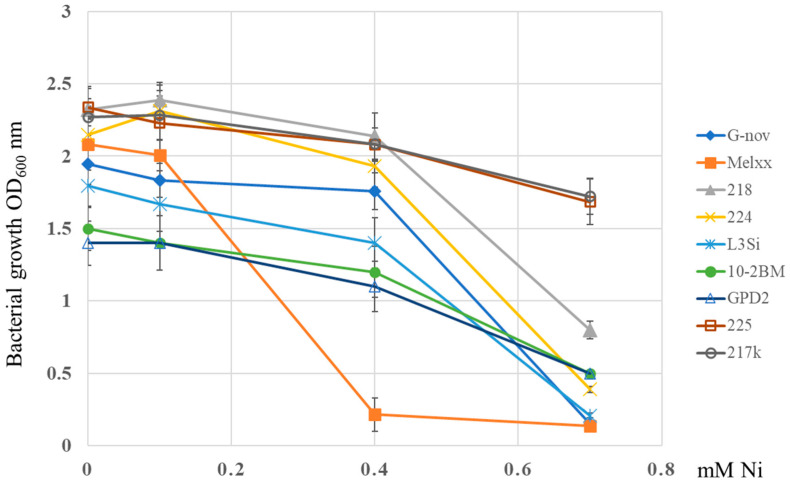
Growth of rhizobial strains (218, 217k, GPD2, 4148ss, 10-2BM, 225, L3Si, Melxx, 4193 cs, G-nov, 224) in the conditions of increasing Ni concentration. The data represent mean ± SD.

We evaluated the growth of 49 strains in a YMA medium supplemented with Ni (Table S1). Screening alfalfa rhizobial strains for nickel tolerance showed that all of the tested strains could grow well in the medium supplemented with 0.1 mM Ni, while seven of the tested strains could tolerate up to 1.2 mM Ni (15% of strains), which is the highest Ni concentration tested in this study (Figure 1, Table S1). In the liquid medium, most strains can grow up to 0.4 mM Ni without a significant reduction (Figure 2) (except the Melxx strain). At the highest tested concentrations of 0.7 mM Ni, the reduction was lowest for strain 217k, OD was reduced by 24% compared to the no-Ni-supplementation culture (Figure 2).

### 3.2. IAA Production by Rhizobium Strains

**Figure 3:**
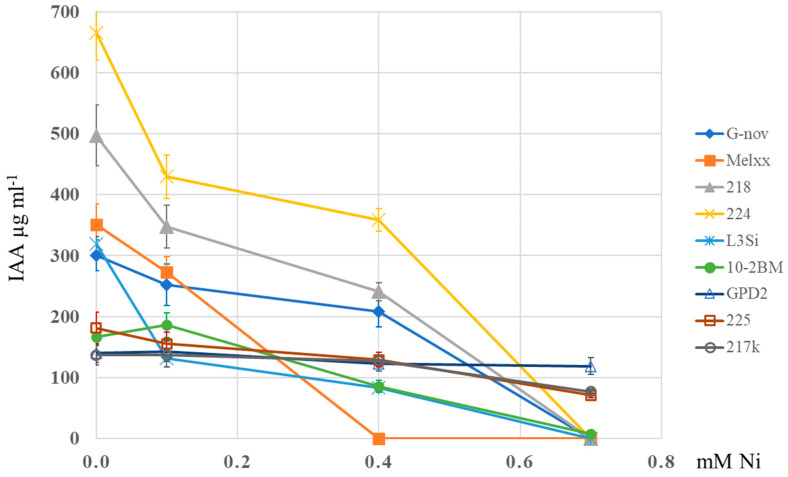
Production of IAA by rhizobial strains (218, 217k, GPD2, 4148ss, 10-2BM, 225, L3Si, Melxx, 4193cs, G-nov, 224) in the conditions of increasing Ni concentration. The data represent mean ± SD.

The good growth of selected strains (Figure 2) at 0.4 mM Ni, both on agar plates and in a liquid medium, indicated the possibility of their further application in the IAA production test. Almost all strains produced high concentrations of IAA when grown in the medium supplemented with tryptophan (2 mg ml^−1^), as a precursor for IAA, without Ni (Table S1). Generally, the addition of Ni in the range from 0.1 to 0.4 mM affected the production of IAA in the same medium with tryptophan (2 mg ml^−1^), but the IAA levels still remained very high (Figure 3). IAA was not detected at 0.7 mM Ni except for strains GPD2, 217k, and 225. For these three strains, the IAA production was retained at the initial level, probably because the growth was not reduced, and there were high correlation coefficients between the OD and IAA concentrations at higher Ni levels (r = −0.051 at 0 mM Ni, r = 0.443 at 0.1 mM Ni, r = 0.670 at 0.04 mM Ni, r = 0.946 at 0.7 mM Ni). Some strains with high symbiotic capacity and IAA production were sensitive to Ni, indicating the importance of metal tolerance evaluation.

### 3.3. ACC Deaminase Activity in Rhizobium Strains

After 7 days of incubation on Petri dishes containing DF medium, the growth of the 218, 217k, Melxx, 4148 ss, L3Si, G-nov, and 225 strains indicated ACC deaminase activity in these strains. ACC deaminase activity was not detected in 10-2BM, 224, and GPD2.

### 3.4. Biosorption of Ni by Rhizobium Strains

**Figure 4:**
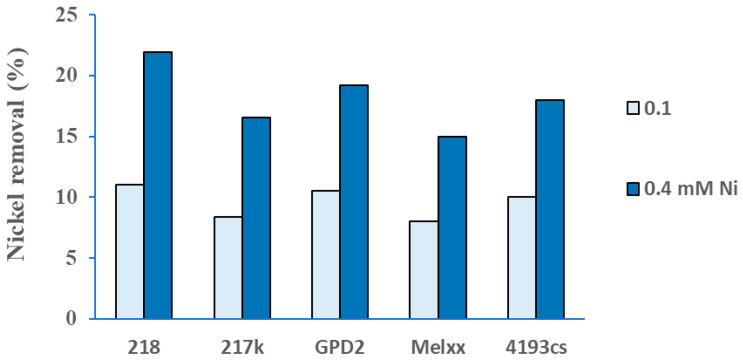
Percentage of Ni removal by rhizobial strains (218, 217k, GPD2, Melxx, 4193cs) from the YMB medium with 0.1 or 0.4 mM Ni after 72 h of incubation at 28 °C and 150 rpm.

The selected IAA-producing Ni-tolerant rhizobial strains were further investigated in nickel biosorption assays. All of the tested strains expressed quite uniform biosorption ability. The strain 218 showed the highest biosorption potential among the strains tested, with a Ni reduction of 21% at 0.4 mM Ni solution and 11% at 0.1 mM Ni after 72 h of growth (Figure 4). The percentage of biosorption of Ni was about 20% higher at 0.4 mM than at 0.1 mM for all tested strains. In this investigation, the biosorption of Ni by the studied rhizobium strains followed the descending order of 218 > GPD2 > 4193cs> 217k> Melxx (Figure 4).

### 3.5. Effect of Ni on Alfalfa Seed Germination

The germination rate of alfalfa seeds in plates with Ni was not decreased up to 0.05 mM of Ni (100% germination), while at the concentration of 0.1 mM, the germination rate was significantly reduced along with the length of the 10-day-old seedlings (Table 3).

### 3.6. Effect of Ni on Growth of Alfalfa Plants Inoculated with Rhizobial Strains

Screening in a standard assay for an alfalfa symbiotic nitrogen fixation test in tubes with Jensen medium (Vincent 1970) confirmed the results from Petri dishes that the germination rate was not influenced up to 0.4 mM Ni, as well as the early growth of seedlings up to 10 days when the negative effect of Ni was not noticeable. After that period, the plants grown in 0.4 mM dried and yellowed (toxic effect of Ni). This was the case with both control plants (Ø, no inoculation and no N supplementation; NØ, no inoculation and N-supplemented medium). Therefore, the growth of alfalfa plants with and without rhizobial inoculation was tested up to 0.1 mM Ni. The strains for inoculation were selected according to Ni tolerance, prominent PGP traits, and previously known symbiotic potential [21]. The results showed that Ni concentrations, inoculation with rhizobial strains, and their interaction affected the alfalfa growth parameters significantly (Table 4). The inoculation with rhizobial strains 218 and 217k induced nodules up to 0.05 mM Ni. However, there were no significant differences in nodule number only between plants grown in 0 and 0.005 mM Ni (Table 4). Although nodules were present at higher Ni concentrations, they were not present in all plants of one treatment, and consequently, the average values of the tested plant parameters were already significantly reduced at 0.01 mM Ni. The Ni at a concentration of 0.01 mM significantly and rapidly reduced the growth of inoculated plants compared to inoculated plants without Ni, and the values for plant parameters (plant height, root length, SDW, and RDW) were similar to those for the control without inoculation and N supplementation (Ø). Plants grown with N supplementation and Ni (NØ) could grow very well in a higher Ni concentration, that is, 0.01 mM, while at the 0.03 mM Ni concentration, growth was rapidly reduced to the level observed without N supplementation. Nitrogen supplementation of a one-third dose of nitrogen in NØ was almost as efficient in plant growth stimulation as a full dose. The SDW is considered as the most valuable indicator of plant growth. The increase in SDW in the presence of Ni varied from 167% for strain 218 at 0.005 mM Ni to 296% for N-supplemented (NØ) plants at 0.005 mM Ni compared to the control plants without N and inoculation (Ø) (Figure S1).

**Figure 5:**
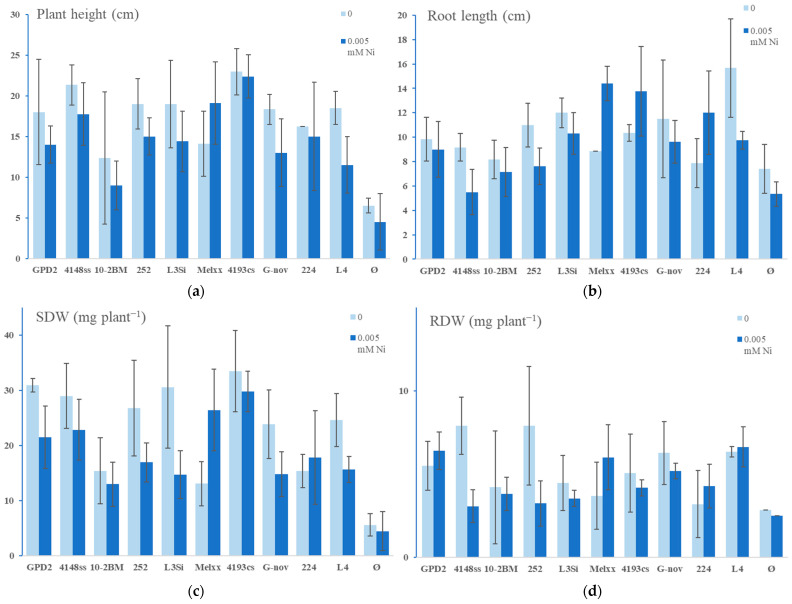
Plant growth parameters of alfalfa in the absence and presence of Ni and different rhizobial strains (GPD2, 4148ss, 10-2BM, 252, L3Si, Melxx, 4193cs, G-nov, 224, L4): (**a**) alfalfa plant height; (**b**) alfalfa root length; (**c**) alfalfa shoot dry weight (SDW); (**d**) alfalfa root dry weight (RDW). Ø—non-inoculated control (no inoculation and no N supplementation). The data represent mean ± SD.

**Figure 6:**
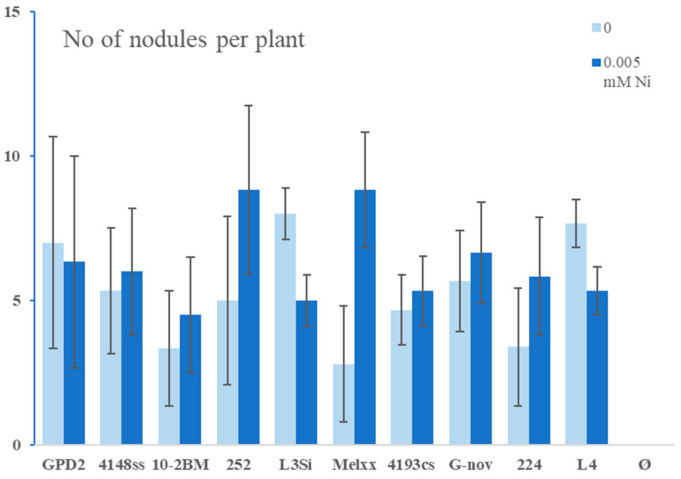
Numbers of nodules in alfalfa in the absence and presence of Ni and different rhizobial strains (GPD2, 4148ss, 10-2BM, 252, L3Si, Melxx, 4193cs, G-nov, 224, L4). Ø—non-inoculated control (no inoculation and no N supplementation). The data represent mean ± SD.

In further testing, 10 additional rhizobial strains were screened for the effect on plants at 0.005 mM Ni. A significant increase in all tested parameters was observed in all inoculated treatments with Ni, compared to non-inoculated plants with Ni (Figure 5 and Figure 6). In addition, inoculation with some strains in the presence of Ni increased the plant parameters compared to inoculated treatments without Ni (for example, strain Melxx increased plant height, SDW, and nodule number over inoculation without Ni).

### 3.7. The Genes Coding for Metal Resistance

The strains were screened for the presence of operons encoding for the trace-metal-resistance determinants, *chrB, ncc,* and *smt*, and the results are shown in Table 5. None of the tested strains possessed *smtA* encoding metal-binding metallothionein or the *chrB* gene encoding a chromate-sensitive regulator of the *chr* operon. The *ncc* operon was amplified as a 1141 bp fragment that spans two genes, *nccA* and *nccN*, and was detected in the strains Melxx and 217k. The presence of *acdS*, the ACC deaminase gene, was determined in 50% of the tested strains (218, 225, GPD2, G-nov). Although ACC deaminase activity was detected in the strains 217k, Melxx, and 4148 ss, the *acdS* gene was not present. In contrast, in the strain GPD2, only the PCR product was detected, but no ACC deaminase activity (Table 5).

### 3.8. Phylogenetic Analysis of Strains

**Figure 7:**
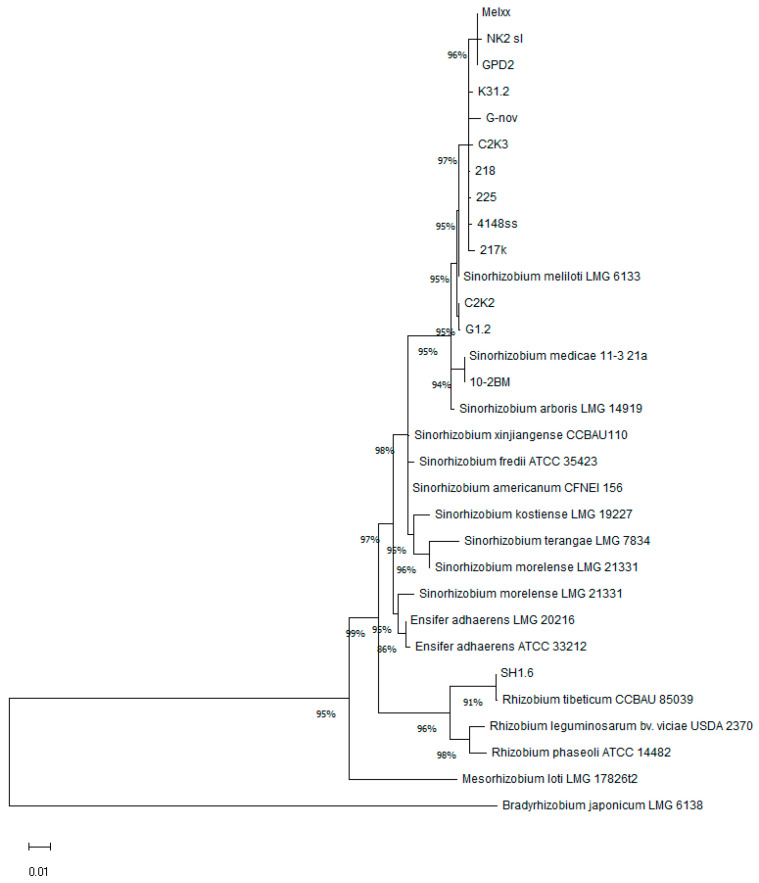
Maximum likelihood tree based on the 16S rRNA gene sequences of the characterized strains and phylogenetically related species. Bootstrap values after 1000 replicates are expressed as percentages. The species *Bradyrhizobium japonicum* is included as the outgroup. The scale bar indicates the fraction of substitutions per site.

The results of the phylogenetic analysis of 16S rRNA gene sequences for the most performant strains and new isolates in this study (in total, 14 strains), together with the reference sequences from GenBank, are shown in Figure 7. The majority of strains, including 218, 225, 4148ss, 217k, Melxx, G-nov, GPD2, NK2 sl, K31.2, C2K3, C2K2, G1.2, and 10-2BM, were affiliated with the genus *Sinorhizobium*. All of the strains (except 10-2BM) grouped together with *S. meliloti* strain LMG 6133 (99.85–100% similarity), while 10-2BM grouped together with *S. medicae* strain 11-3 21a (100% similarity). In addition, one strain, SH1.6, was affiliated with the genus *Rhizobium*, with *R. tibeticum* CCBAU 85039 as the closest type strain (99.92% similarity).

## 4. Discussion

### 4.1. Tolerance of Rhizobia to Nickel

Previous studies indicated that the effect of rhizobial inoculation on trace metal accumulation in legumes is dependent on growth promotion and phytoavailability of trace metals [31]. Rhizobium should have metal resistance to improve legume–rhizobium symbiosis in the bioremediation of metal-polluted soil [32]. Therefore, the selection of metal-tolerant rhizobia is the first step in implementing inoculation practices since the rhizobium is considered relatively sensitive to metals [33]. The strain tested in this study can be designated as exhibiting medium tolerance to Ni with respect to previous studies of *Sinorhizobium* species (tolerant mostly to 1 mM Ni and 3 mM) [34] and other rhizobia (tolerant in the wide range from about 0.2 mM Ni to 1000 µg g^−1^) [18,19,35]. The strains with higher tolerance are mostly isolated from soils with higher Ni concentrations, like serpentine soils [35]. However, compared to the genera different from the rhizobia, the tested Ni concentrations in our study were relatively low. For comparison, the highest tolerated concentration by bacteria isolated from serpentine soils was as high as 64 mM for isolates belonging to *Streptomyces* and *Nocardia* genera, and all of the isolates tolerated at least 1 mM [36].

### 4.2. Plant Growth-Promoting Traits of Rhizobia

Legume growth could be stimulated through general mechanisms of plant growth promotion such as IAA, siderophores, and ACC deaminase production [37,38]. All of these PGP mechanisms have been recognized in the group of rhizobia. Indole-3-acetic acid is involved in cell division and enlargement, tissue differentiation, regulation of root system architecture, controlling primary root elongation and lateral root formation, and resistance to stressful conditions [39,40]. In our study, all strains could produce IAA in high concentrations in the presence of Ni. Similarly, significant IAA production by rhizobial strains under Ni conditions was detected in *Rhizobium* and *Mesorhizobium* [35,41]. The production of IAA could be essential for plant growth promotion under metal stress since IAA mainly helps plants by increasing the acquisition of nutrients through increased root development [37,42].

Biosorption (the absorption of substances by microbial biomass) has been recognized as one of the most efficient mechanisms of trace metal removal by rhizobia and bacteria in general [15]. In particular, rhizobia produce a great amount of exopolysaccharide (EPS) that can bind metals through electrostatic interactions [43,44]. Similarly to previous research, the higher removal of Ni from aqueous solution was detected at higher Ni concentrations. Biosorption of Ni was significantly higher at the rising concentrations examined as compared to the lower concentration of Ni and increased with increasing concentration [19]. The *Sinorhizobium* strain BEL5B showing an enhanced exopolysaccharide production under nickel stress showed the highest nickel tolerance and a good potential for nickel biosorption. The functional groups such as hydroxyl and the amino groups present on the lipo- and exopolysaccharide layers of the cell surface were involved in the biosorption of nickel [34].

In the nickel-resistant rhizobia, the *ncc* operon is responsible for the high level of combined nickel, cobalt, and cadmium resistance, and the *nre* for the low level of nickel resistance [45,46]. *nccA*, encoding a nickel–cobalt–cadmium resistance protein, a component of the cation efflux system (*nccYXHCBAN*), forms a membrane tunnel through which ion transport occurs [47,48]. In our study, the *nccA* gene component of the *ncc* operon was detected in only two out of eight tested isolates showing Ni tolerance and the ability of normal growth in media supplemented with Ni (1.2 mM), suggesting that some other genes and mechanisms are involved in Ni tolerance. Concentrations higher than 1.2 mM were not tested in this study; therefore, tolerance to even higher concentrations is not excluded. In the study of Jobby et al. [34], the *ncc* operon in *Sinorhizobium* strain BEL5B was not also detected. This is the strain that showed the highest nickel tolerance, and it was hypothesized that this could be due to the divergence of the BEL5B operon in the primer binding site or a different order of the genes. Schmidt and Schlegel [48] showed that this resistance complex probably works as a cation proton antiporter and is composed of a regulatory gene region *nccYXH* followed by the structural region *nccCBA*. In addition, other operons known to be involved in nickel tolerance in rhizobia include *nre* (nickel resistance), *cnr* (cobalt–nickel resistance), and *ncc* (nickel–cobalt–cadmium resistance) genes [45]. The *nreB* gene encodes a gene for a Ni^2+^/H^+^ antiporter. In a study by Pini et al. [49], phylogenetic analysis of *nreB* orthologs suggested multiple horizontal gene transfer events in the past. All of the sequenced *S. meliloti* genomes carried the gene SMa1641, which encodes a nickel/proton antiporter, homologous to *nreB* present in *Cupriavidus metallidurans* 31A. Since this gene is located on the megaplasmid pSymA, required for symbiosis, its role in symbiotic interactions was suggested [49]. We assume that our *Sinorhizobium* strains may also have the *nreB* gene. It is widely accepted that five mechanisms play a role in metal tolerance in bacteria: (1) metal exclusion; (2) active transport outside of the cells; (3) intracellular or extracellular sequestration; (4) enzymatic conversion to a less toxic form; (5) lower sensitivity of cellular targets [36,50].

ACC (1-aminocyclopropane-1-carboxylate) is a precursor of the plant hormone ethylene, involved in many biochemical processes in plants, including the response to environmental stresses, such as drought and trace metal toxicity [51,52]. The ACC deaminase gene, *acdS*, has been identified in several rhizobial species, such as *R. leguminosarum bv. trifoli* and *Mesorhizobium loti*, included in the alleviation of trace metal stress [53]. In our study, the ACC deaminase gene was determined in half of the strains tested. In addition, the ACC deaminase activity was detected in strains with the acdS gene (except one strain), as well as in the strains where the gene was not detected. Normally, ethylene in small amounts can have a protective role. However, an increase in ethylene concentration, which occurs under stressful biotic or abiotic conditions, can cause negative effects such as inhibition of plant growth, senescence, abscission, chlorosis, defoliation, and disturbed root development. The enzyme ACC deaminase catalyzes the breakdown of ACC into α-ketobutyrate and ammonia, and by taking up some of the ACC exuded from the seeds or roots, it lowers the level of ethylene in plants and consequently mitigates the negative effects caused by it. The role of ACC deaminase is especially interesting in rhizobial species since ethylene inhibits nodulation by rhizobia in various legume species [51,52]. Genetically engineered *Sinorhizobium meliloti* with an introduced ACC deaminase structural gene (*acdS*) and its upstream regulatory gene, a leucine-responsive regulatory protein (LRP)-like gene (*lrpL*), from *R. leguminosarum bv. viciae* 128C53K, formed 35 to 40% more nodules and also increased shoot dry weight by approximately 33% in alfalfa plants, compared to the same strain which does not produce ACC deaminase [54]. Previously, the same author showed that two *R. leguminosarum bv. viciae* 128C53K insertion mutants in the abovementioned genes acdS and lrpL, neither of which synthesized ACC deaminase, were less effective in nodulating *Pisum sativum* L. cv. Sparkle [55].

### 4.3. Growth of Alfalfa Affected by Nickel and Rhizobia

In general, there is a reduction in seed germination as Ni concentrations increase in the growing media. Previously, 20 ppm of Ni reduced seed germination significantly, while seed germination and plant growth were seriously affected by 40 ppm of Ni after two weeks of exposure to trace metals (24% reduction) [56]. Similar results were obtained by Peralta et al. [57] for the same medium and period of seedling growth (25% seed germination reduction), while the seedling lengths were reduced by 58% at 40 ppm of Ni. In our research, a significant reduction in seed germination of 15% was detected for 0.5 mM Ni, while the seedling lengths were reduced already at 0.1 mM Ni. In prolonged cultivation of plants for up to 40 days, it was found that the much lower Ni concentrations of 0.03 mM influenced plant growth, namely plants were drying up in both types of media with nitrogen and without nitrogen supplementation, indicating the limitation of short germination tests.

Trace metals, including Ni, have a great influence on nodulation [58]. Excess Ni has shown deleterious effects on the genus *Rhizobium* and, hence, on nodule formation in several leguminous species [59]. In our study, nodulation was evident up to 0.05 mM inoculation, but the nodules were inactive since the plants were small and yellow and at the level of non-inoculated control. At the concentrations of 0.005 mM Ni, plants formed nodules with all 12 investigated alfalfa rhizobial strains, and all plants formed active symbiosis indicated by high green plants, with increased SDW compared to non-inoculated control with and without Ni. Some strains like Melxx exhibited higher plant height, root length, SDW, and RDW as well as nodule number at 0.005 mM Ni. Similarly, the strain 217k resulted in a higher SDW in the presence of low Ni concentrations. The plants associated with *Rhizobium* sp. HGR-4 showed higher nodulation, nitrogen level, and leghemoglobin content in soils amended with 80 mg g^−1^ Ni than the control plants without HGR-4 inoculation in horse gram [18]. In addition, inoculation with the most tolerant *Rhizobium* TAL–1148 + *B. subtilis* treatment was shown to be more effective in terms of growth parameters (dry weight of plant, plant height, number of nodules, and N_2_ content) in *Vicia faba* in artificially contaminated soil with under 600 mg kg^−1^ Ni stress conditions [19]. The symbiotic efficiency including nodulation and nitrogen fixation abilities of metal-tolerant strains of *R. leguminosarum* and *S. meliloti* was previously reported [60]. Some studies have shown the importance of rhizobia in mitigating Cu, Cd, and other trace metal stress in the alfalfa [60,61,62]. This work indicates the possibility of using *Sinorhizobium* strains to promote growth in alfalfa plants grown in the presence of elevated Ni contents. Some rhizobia, having symbiotic efficiency, plant growth-promoting capacity, and resistance to nickel, indicate that there are abundant functional microbial resources in the soil environment for further improvement of alfalfa plant tolerance in soils with increased trace metals.

## 5. Conclusions

It is considered that the use of metal-tolerant rhizobial inoculants can be an effective measure for mitigating metal stress and enabling the promotion of the growth of plants in soils with elevated metal concentrations. This study reveals native nickel-tolerant alfalfa-associated rhizobia with high symbiotic potential. Most of the strains maintain their plant growth-promoting traits under nickel stress; that is, they can produce IAA. The potential of nickel removal by rhizobia up to some extent from the medium was also detected. Under different nickel concentrations, the growth of alfalfa plants was reduced either in inoculation or nitrogen supplementation, indicating that elevated Ni levels can considerably impair plant health. Compared to the control plants without rhizobial inoculation, *Sinorhizobium* spp. strains effectively promote plant growth in the presence of nickel at lower concentrations. The nodulation was retained, and the plant parameters were improved. The most performant strains were selected to study the growth-promoting properties in the soil conditions. This study contributes to the advancements in the sustainable use of contaminated lands; however, this was a first step in the development of an efficient inoculant, and further research is necessary to refine strain selection and application techniques in metal-burdened soils.

## Figures and Tables

**Table 1:** List of primers used in this study.

Primer Sequence	Gene	Primer Name
5′-AGAGTTTGATCMTGGCTCAG-3′ 5′-TACGGYTACCTTGTTACGACTT-3′	16S rRNA	27F 1492R
5′-GGATTAGATACCCTGGTA-3′ 5′-CCGTCAATTCMTTTRAGTTT-3′	16S rRNA	785F 907R
5′-GGCAAGGTCGACATCTATGC-3′ 5′-GGCTTGCCATTCAGCTATG-3′	*acdS*	Acds F Acds R
5′-GATCGACGTTGCAGAGACAG-3′ 5′-GATCGAGGGCGTTTTGATAA-3′	*smtA*	smtA F smtA R
5′-GTCGTTAGCTTGCCAACATC-3′ 5′-CGGAAAGCAAGATGTCGATCG-3′	*chrB*	chrB F chrB R
5′-ACGCCGGACATCACGAACAAG-3′ 5′-CCAGCGCACCGAGACTCATCA-3′	*nccA*	nccA F nccA R

Y = C/T; R = A/G; M=C/T.

**Table 2:** PCR conditions.

	*nccA*	*chrB*	*smtA*	*acdS*
Initial denaturation	95 °C, 5 min	95 °C, 5 min	95 °C, 5 min	94 °C, 3 min
Denaturation Annealing Extension	95 °C, 30 s 58 °C, 1 min 72 °C, 90 s	94 °C, 1 min 60 °C, 30 s 72 °C, 2 min	94 °C, 1 min 56 °C, 1 min 72 °C, 1 min	94 °C, 1 min 62 °C, 1 min 72 °C, 3 min
Final extension	72 °C, 5 min	72 °C, 5 min	72 °C, 5 min	72 °C, 5 min
Number of cycles	35	30	35	35

**Table 3:** Germination of alfalfa seeds in the presence of Ni.

Concentration of Ni (mM)	0	0.005	0.01	0.03	0.05	0.1	0.5	0.8	1.0
Germination rate (%)	100	100	100	100	100	98.12	87.56	77.19	74.26
Seedlings length (cm)	7.08 a	6.78 a	6.82 a	6.46 ab	6.55 ab	6.13 b	3.03 c	2.12 d	1.75 d ^1^

^1^ The means in the same row followed by the same letter are not significantly different at *p* < 0.05.

**Table 4:** Plant growth parameters of alfalfa in the presence of Ni and rhizobial inoculation.

Treatment	Ni mM	Shoot Height cm	Root Length cm	Nodule Number	SDW mg Plant^−1^	RDW mg Plant^−1^
218	0	19.67 a	14.62 a	5.71 a	19.63 a	2.34 ab
	0.005	13.44 b	11.17 b	4.43 ab	16.44 a	3.74 a
	0.01	5.67 c	4.57 c	1.71 bc	4.44 b	2.23 bc
	0.03	4.54 c	3.69 c	0.71 c	4.34 b	1.91 bc
	0.05	3.31 c	3.43 c	0.29 c	4.40 b	1.63 b
	0.10	3.87 c	1.46 d	0.00 c	2.20 b	0.63 c
217k	0	15.20 a	11.16 a	6.85 a	14.46 b	3.68 b
	0.005	16.57a	9.14 a	5.00 a	19.57 a	4.50 ab
	0.01	5.23 b	5.21 b	2.29 b	5.19 c	2.89 bc
	0.03	4.56 b	4.20 bc	2.33 b	3.04 c	1.59 cd
	0.05	3.97 b	2.54 c	0.57 b	4.97 c	1.83 de
	0.10	3.30 b	1.20 d	0.00 b	3.38 c	0.74 de
Ø	0	6.50 a	7.39 a	0.00 ns	5.64 a	2.83 ns
	0.005	4.50 b	5.35 bc	0.00	4.48 ab	2.50
	0.01	6.51 a	6.89 bc	0.00	5.21 ab	3.19
	0.03	4.43 b	4.21 bc	0.00	5.16 ab	3.51
	0.05	3.63 c	5.13 bc	0.00	3.25 b	1.89
	0.10	2.92 c	1.14 c	0.00	3.33 b	0.75
NØ	0	16.93 a	11.08 a	0.00 ns	29.50 a	5.96 a
	0.005	19.17 a	10.21 a	0.00	22.24 a	3.90 b
	0.01	16.71 a	10.36 a	0.00	24.73 a	5.30 b
	0.03	4.17b	2.36 b	0.00	5.84 b	1.59 b
Source of variation						
Ni mM		⁎⁎⁎	⁎⁎⁎	⁎⁎⁎	⁎⁎⁎	⁎⁎⁎
Treatment		⁎⁎⁎	⁎⁎⁎	⁎⁎⁎	⁎⁎⁎	⁎⁎⁎
Interaction		⁎⁎⁎	⁎⁎⁎	⁎⁎⁎	⁎⁎⁎	⁎⁎⁎

*** Significant at the 0.001 probability level. ns—statistically not significant. The data represent the mean, and values followed by the same letter in the column are not significantly different according to Duncan’s test at *p* < 0.05 for treatment. Rhizobial strains—*Sinorhizobium meliloti* strains 218, 217k. Ø—non-inoculated control (no inoculation and no N supplementation). NØ—treatment with N supplementation and without inoculation.

**Table 5:** Presence of genes for trace metal tolerance and ACC in alfalfa rhizobial strains.

Rhizobia Strains	*smtA*	*chrB*	*nccA*	*acdS*
218	nd	−	−	+
217k	nd	−	+	−
G-nov	nd	nd	−	+
GPD2	−	−	−	+
225	−	−	−	+
4148ss	−	−	−	−
Melxx	−	−	+	−
10-2BM	−	−	−	−

nd—gene not determined; −—gene not detected; +—gene detected.

## Data Availability

All raw sequence data have been made available in the NCBI Sequence database under the following accession numbers: PQ788852, PQ788854, PQ788857, PQ788858, PQ788859, PQ788862, PQ788863, PQ788864, PQ789180, PQ789181, PQ789184, PQ789185, PQ789231, PQ789240, PQ789241.

## Supplementary Materials

The following supporting information can be downloaded at: https://www.mdpi.com/article/10.3390/microorganisms13020340/s1: Table S1: Strains from alfalfa nodules, origin, identification, and tolerance to Ni. Figure S1: Percentage of SDW increase compared to the control non-inoculated plants (Ø).
