# Supplementary material for: The Plant Growth-Promoting Ability of Alfalfa Rhizobial Strains Under Nickel Stress
Source: Microorganisms. 2025 Feb 5;13(2):340. doi: 10.3390/microorganisms13020340 (PMC11858492; doi:10.3390/microorganisms13020340)
Supplement: Supplementary file 1 [file microorganisms-13-00340-s001.zip › microorganisms-3415316-supplementary.pdf]

Supplementary Table S1. Strains from alfalfa nodules, origin, identification and tolerance to Ni.

No	Strain	Origin	Ni tolerance mM	IAA production µg ml^{-1}	Identification
1	201k	*	0.90	>200	*S. meliloti**
2	202i	*	1.00	>200	
3	203	*	0	>200	*S. meliloti**
4	204	*	0.90	50	
5	205	*	1.00	>200	*S. meliloti**
6	206k	*	0.10	>200	*S. meliloti**
7	207	*	0.10	>200	*S. meliloti**
8	208k	*	1.20**	>200	*S. meliloti**
9	209k	*	1.10	>200	
10	211-1	*	1.00	>200	
11	212	*	0.10	>200	
12	215k	*	0.10	>200	
13	217k	*	1.10	>200	*S. meliloti*
14	218	*	1.20**	>200	*S. meliloti*
15	221i	*	0.80	>200	*S. meliloti**
16	222k	*	1.00	>200	
17	223bk	*	1.00	>200	
18	224	*	1.10	>200	*S. meliloti**
19	225	*	1.00	>200	*S. meliloti*
20	226	*	1.10	>150	
21	231	*	0.10	>200	
22	232	*	0.80	>200	
23	237n	*	1.10	15	*S. meliloti**
24	238	*	1.10	>200	
25	240n	*	0.80	>200	
26	241sk	*	0.50	>200	
27	247	*	0.70**	>200	*S. meliloti* *
28	249-345	*	0.70**	nd	
29	250	*	0.50	>200	*S. meliloti**
30	252-345	*	0.70**	>200	*S. meliloti**
31	L3Si	*	0.50	>100	*S. meliloti**
32	L4	*	0.40	>200	*S. meliloti**
33	L5	*	0.40	>200	
34	4148ss	*	0.80	150	*S. meliloti*
35	4148pd	*	1.20**	>200	
36	4193cs	*	1.20**	>200	*S. meliloti**
37	Melxx	*	0.90	>200	*S. meliloti*
38	G-nov	This work	1.10**	>200	*S. meliloti*
39	10-2BM	This work	1.20	100	*S. medicae*
40	G-V2-1	This work	1.20	150	
41	10-2N	This work	0.80	150	
42	GPD2	This work	1.20**	150	*S. meliloti*

43	C2K2	This work	0.70	150	*S. meliloti*
44	C2K3	This work	0.80	150	*S. meliloti*
45	G1.2	This work	0.40	150	*S. meliloti*
46	C1-1.1	This work	0.80**	150	*S. meliloti*
47	K31.2	This work	0.80**	150	*S. meliloti*
48	NK2 sl	This work	0.80	150	*S. meliloti*
49	SH1.6	This work	0.70	150	*R.tibeticum*

* The origin and/or identification according to Stajković-Srbinović et al. [21]. **not tested at higher concentrations

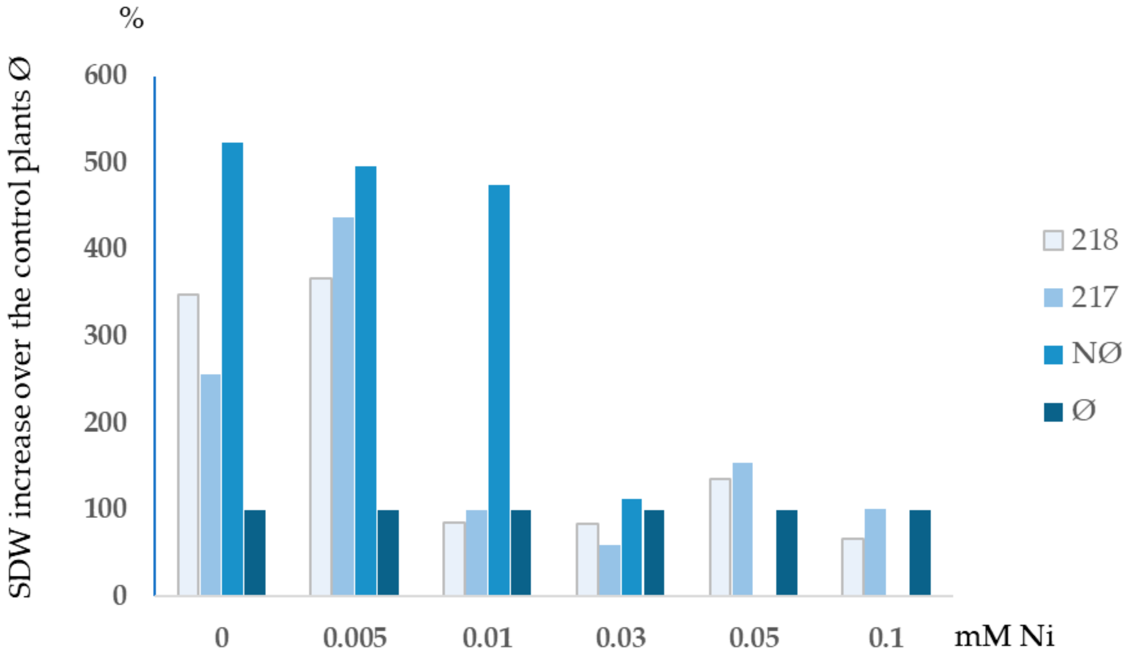

Supplementary Figure S1. Percentage of SDW (shoot dry weight) increase compared to the control non-inoculated plants (Ø). Rhizobial strains - *Sinorhizobium meliloti* strains 218 and 217k. Ø - Non-inoculated control (no inoculation and no N supplementation). NØ - Treatment with N supplementation and without inoculation. The SDW of the control non-inoculated plants (Ø) was designated as 100%.
